# Health Insurance Literacy and Medical Debt in Middle-Age Americans

**DOI:** 10.3928/24748307-20211102-01

**Published:** 2021-10

**Authors:** Jacqueline Wiltshire, Echu Liu, Caress A. Dean, Edlin Garcia Colato, Keith Elder

## Abstract

**Background:**

Health insurance literacy (HIL) may influence medical financial burden among people who are sick and the most vulnerable.

**Objective:**

This study examined the relationships between HIL, health insurance factors, and medical debt among middle-age Americans, a population with an increasing prevalence of illnesses.

**Methods:**

Linear and generalized linear regression analyses were conducted on data drawn from the 2015–2016 waves of the Health Reform Monitoring Survey, a national, internet-based sample of Americans age 18 to 64 years. The analytical sample included 8,042 people age 50 to 64 years.

**Key Results:**

Adjusted mean HIL scores did not differ by private versus public insurance or by out-of-pocket costs. Mean HIL scores were lower with higher deductibles; however, differences in mean scores were small. Higher HIL was associated with lower medical debt (odds ratio = 0.97; 95% confidence interval [0.96, 0.98]), but at the highest HIL score, the risk of having medical debt was still 13.8%. Public coverage, higher annual deductibles, and out-of-pocket costs were associated with higher risks of having medical debt.

**Conclusions:**

The findings suggest that HIL plays an important role in medical debt burden. However, with the shift toward high cost-sharing insurance plans, addressing health care affordability issues along with HIL are critical to eliminate medical debt problems. **[*HLRP: Health Literacy Research and Practice*. 2021;5(4):e319–e332.]**

**Plain Language Summary:**

Understanding and using health insurance (also defined as health insurance literacy) may influence the ability to pay medical bills among people who are sick and vulnerable. This study examined the relationships among health insurance literacy, health insurance factors, and difficulty paying medical bills (i.e., medical debt) in Americans age 50 to 64 years using data from the Health Reform Monitoring Survey. People with higher health insurance literacy reported lower medical debt. Type of insurance coverage did not influence medical debt. Those with annual deductibles and out-of-pocket health care costs were more likely to report having medical debt.

Rising health care costs and declining insurance benefits have made medical bills and debt a major problem for middle-age Americans age 50 to 64 years (Komisar, 2012; Zilcha & Schneier, 2012). In a 2018 representative survey of middle-age Americans, 27.4% reported having low confidence in their ability to pay for health insurance and out-of-pocket costs in the coming year (Tipirneni et al., 2020). Americans ration and forgo necessary care, experience significant financial hardships, and even file for bankruptcy because of medical debt (Doty et al., 2008; Himmelstein et al., 2009; Komisar, 2012, 2013). Middle-age Americans are at the greatest risk of medical bills and debt due to their increasing prevalence of illnesses and limited or decreasing financial resources to pay out-of-pocket costs (Chen et al., 2004; Tu & Liebhaber, 2009; Yee et al., 2012). Many leave health needs untreated until they enter Medicare, which increases the program's annual costs (Chen et al., 2004; Nadash et al., 2018).

The 2010 Affordable Care Act (ACA) expanded access to insurance coverage for Americans age 18 to 64 years, which provided coverage options to middle-age Americans who previously had no options for coverage before becoming eligible for Medicare (Collins, Bhupal, et al., 2019; Jones et al., 2018; Yee et al., 2012). More people are insured since the ACA was enacted, but many people are also now insured in plans with high deductibles and other cost-sharing features (Collins, Bhupal, et al., 2019), which increase vulnerability to high medical bills and debt (Collins, Bhupal, et al., 2019; Galbraith et al., 2011; Rabin et al., 2020; Zhang et al., 2018). High cost-sharing plans are designed to make people assume responsibility for making cost-conscious decisions about their health care use (Kullgren et al., 2010; Zhang et al., 2018). However, studies show that many people do not understand the benefits and cost-sharing conditions of their health insurance plans (Bartholomae et al., 2016; Loewenstein et al., 2013; Reed et al., 2012). They also do not understand basic insurance terms such as premium, deductible, copayments, coinsurance, and maximum out-of-pocket (Bartholomae et al., 2016; Loewenstein et al., 2013). Loewenstein et al. (2013), using data from two surveys of representative samples of Americans with private health insurance, showed that only 14% of respondents correctly understood basic insurance terms. The proportion of people in high and complex cost-sharing plans has steadily increased (Collins, Bhupal, et al., 2019), but little attention has been placed on their ability to understand and use their health insurance (Paez et al., 2014; Tipirneni et al., 2018). This suggests that increasing numbers of insured people have difficulty making informed or optimal cost-conscious decisions about their health care.

The ability to understand, select, and use health insurance is defined as health insurance literacy (HIL) (Loewenstein et al., 2013; Paez et al., 2014). The ACA has underscored the lack of HIL, especially among the most vulnerable populations (Paez et al., 2014). HIL has also been shown to be lower among people with Medicare or Medicaid compared to those with employer-sponsored insurance (Kutner et al., 2006; Loewenstein et al., 2013). Research also shows that HIL is universally low (Loewenstein et al., 2013; Long et al., 2014), especially among people with lower education, lower income, and poorer health (Long et al., 2014; McCormack et al., 2009).

HIL influences health care decision-making and use of preventive and nonpreventive health services **(**Morgan et al., 2008; Reed et al., 2012; Tipirneni et al., 2018). Low HIL is associated with greater delay or avoidance of care due to cost, including preventive care (Morgan et al., 2008; Reed et al., 2012; Tipirneni et al., 2018), which has been shown to lead to higher costs and adverse health outcomes downstream (Trivedi et al., 2010; Zheng et al., 2019). A recent study by Tipirneni et al. (2018) showed that a 12-point increase in HIL was associated with a 39% lower likelihood of delayed or for-gone preventive care and a 29% lower likelihood of delayed or forgone nonpreventive care. Other studies have shown that low HIL was associated with rationing of medication due to cost pressures and multiple emergency department visits among Medicare beneficiaries (Morgan et al., 2008; Piette & Heisler, 2006). Less is known about how HIL may influence medical debt, an outcome in health care services use. This study examines the relationships between HIL, health insurance features, and medical debt among middle-age adults, a population group which tends to have high health care needs and incur high health care costs (Chen et al., 2004; Yee et al., 2012).

## Methods

### Data Source and Sample

Our study uses data from the Health Reform Monitoring Survey (HRMS), which was designed to collect information on the ACA's implementation (Long et al., 2014). The HRMS collects information on health insurance coverage, access to care, affordability of care, and health status. Each round of the HRMS is conducted in a random sample of approximately 7,500 people quarterly, who are drawn from a probability-based, nationwide, internet-based panel of 55,000 civilian, noninstitutionalized Americans age 18 to 64 years. The survey sample is representative of Americans who have internet access. As a result, this internet-based sample may not be representative of people with low-income, people who are undereducated, and racial and ethnic minorities. Economic inequality and systemic racial discrimination have contributed to the lack of internet access in marginalized communities (Turner, 2016). Comparison of people age 50 to 64 years in the HRMS with those in Behavioral Risk Factor Surveillance System Surveys (BRFSS), a widely used nationally representative survey, indicates that HRMS has a higher percentage of non-Hispanic White people (76.41% vs. 63.35%) and a lower percentage of people with less than a high school education than the BRFSS (7.04% vs. 14.15%). See the supplemental table for comparisons between HRMS and the BRFSS.

Our analysis was limited to the 2015 and 2016 waves of the HRMS. These waves collected information on health insurance literacy, a key measure in our analysis. The sample was 8,339 people age 50 to 64 years; our analytical sample was limited to 8,042 insured people. Fifty-five people were excluded for being uninsured or missing values on the outcome variable. Also 242 people were excluded because of missing values on the out-of-pocket expenses and deductible variables. These respondents reported that they had out-of-pocket expenses and a deductible but refused to provide the amount. Detailed descriptions of the HRMS and its documentation are available from the HRMS website (http://hrms.urban.org/) (Long et al., 2014). The HRMS receives its core funding from the Robert Wood Johnson Foundation and Urban Institute (http://hrms.urban.org/about.html).

### Conceptual Framework

Andersen's Behavioral Model of Health Services Use (BMHSU), one of the most widely used models to explain medical care use and outcomes, guides the selection of variables to include in our study (Andersen, 1995; Andersen & Newman, 2005). The BMHSU is useful for selecting, identifying, and sequencing variables in the process of health services use (Andersen & Newman, 2005). Previous work has established the value of the BMHSU in understanding the predictors and mediators of medical bill problems (Wiltshire, Elder, & Allison, 2016; Wiltshire, Elder, Kiefe, et al., 2016). The BMHSU posits that peoples' use of health care services and subsequent outcomes are influenced by predisposing, enabling, and need factors. According to the BMHSU, there are existing conditions that are not directly responsible for use of health services that predispose people to use or not use services. Predisposing factors include age, race and ethnicity, marital status, and education. Enabling factors facilitate or impede health services use and include income, insurance status, and having a usual source of care. Need indicates the health of a person who may require medical care (Andersen, 1995). Need is a person's perceived and/or professionally evaluated health status. The relationships between the components of the model (i.e., predisposing, enabling, and need factors; medical care use; and outcomes) are reciprocal.

**Figure 1.**
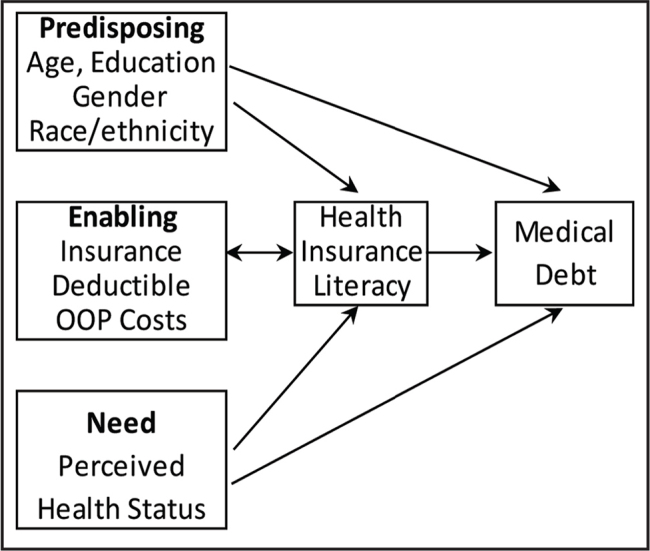
Conceptual framework illustrating relationships between HIL, health insurance factors, and medical debt. Adapted from Andersen & Newman (2005) and Andersen (1995).

The BMHSU suggests medical debt is a function of predisposing, enabling, and need variables (**Figure 1**). Medical debt is incurred through the use of health care services and can be viewed as an outcome of that use. It can also influence health outcomes because it can deter adherence to prescribed treatments and may limit further use of health care services (Collins, Bhupal, et al., 2019; Tipirneni et al., 2020). Consequently, medical debt can be viewed as an important enabling factor of health services use because it reflects people's lack of economic resources (i.e., insurance and income) and inability to pay for health care services (Tipirneni et al., 2020). HIL can be viewed as an enabling factor because it has been shown to facilitate or impede access to and use of care (Tipirneni et al., 2018). Low HIL can lead to poor selection and use of insurance, which can lead to medical debt and poor health outcomes (Loewenstein et al., 2013; Tipirneni et al., 2018). Conversely, people can become knowledgeable about their health insurance coverage from their experiences of dealing with medical debt.

### Dependent Variable

The following item from the HRMS was used to assess medical debt: Do you or anyone in your family currently have any medical bills that are being paid off over time? Responses were coded as yes or no. The words or terms “medical bills that are being paid off over time” and “medical debt” will be used interchangeably.

### Key Independent Variables

***Health insurance literacy.*** We measured HIL, the key independent variable in the analysis, by a composite score, that is, the summation of responses to the following seven questions about health insurance coverage: How confident are you in your ability to understand the definitions of (1) premium, (2) deductible, (3) co-payments, (4) co-insurance, (5) maximum annual out-of-pocket spending, (6) provider network, and (7) covered services? The responses to these seven questions were recorded as 1: *very confident*; 2: *somewhat confident*; 3: *not too confident*; or 4: *not at all confident*. Items were reverse coded so that *very confident* equals 4 and *not at all confident* equals 1. Given the ordinal scale of the response to these questions, a maximum HIL score is 28 and the minimum score is 7. Higher scores indicate higher HIL. The seven items were summed instead of using other calculation methods (e.g., mean confidence score) as the literature suggests there are limitations to placing a number value to Likert-type responses (Sullivan & Artino, 2013).

Cronbach's alpha and factor analysis techniques were used to determine if the seven items were sufficiently intercorrelated and if the grouped items measure health insurance literacy. Item-total correlation ranged between 0.59 and 0.82, and one general component accounted for 38.56% of the variance. The grouped items had an internal consistency of 0.94. These statistics indicate that our grouped items measure health insurance literacy appropriately.

***Health insurance features.*** The majority of the sample was insured (99.61%); therefore, we created an indicator variable for insurance type (private vs. public coverage). As per the HRMS, private coverage included health insurance obtained through a current or former employer or union as well as purchased directly from an insurance company. Public coverage was categorized as people who reported having Medicare, Medicaid, Tricare, or any kind of state- or government-sponsored assistance plan based on income or a disability. In the HRMS public-use dataset, out-of-pocket expenses and deductible variables were top-coded to protect respondents' anonymity and prevent disclosure risk. We created an indicator variable for out-of-pocket expenses (0–$999, $1,000–$2,999, $3,000–$5,999, ≥$6,000) which was generated from the following question: In the past 12 months, about how much have you and your family spent out-of-pocket for health care costs that were not covered by your health insurance or health coverage plan? An indicator variable for deductible (0–$499, $500–$1,499, $1,500–$2,499, $2,500–$3,999, ≥$4,000) was also created based on the following question: What is the annual deductible per person under your health insurance or health coverage plan?

### Control Variables

To appropriately assess the associations among HIL, insurance features, and medical debt, we included several variables known to influence access to and use of health care services (Andersen & Newman, 2005; Andersen, 1995). Previous work has also established these variables as predictors and mediators of medical bill problems (Wiltshire, Elder, & Allison, 2016; Wiltshire, Elder, Kiefe, et al., 2016). Variables included were as follows: race/ethnicity (White non-Hispanic, Black non-Hispanic, Other non-Hispanic, and Hispanic, all mutually exclusive), age (50–54, 55–59, 59–64), gender (male/female), marital status (currently married/not married), and education level (less than high school, high school, some college, Bachelor's degree or higher). Income was assessed as a percent of poverty level (based on the U.S. poverty guidelines): ≤138% of the federal poverty level (FPL), 139% to 399% of the FPL, and ≥400% of the FPL. The FPL categories used by HRMS are also based on ACA premium subsidy eligibility. Having a usual source of care (yes/no) and perceived health status (poor/fair, good, very good, excellent) were also included in our analyses.

### Analysis

Descriptive statistics were reported for all study variables by medical debt (i.e., medical bills that are being paid over time). Statistical significance was based on the Pearson chi-square test for categorical variables and *t*-test for continuous variables. We used linear regression models to assess differences in HIL mean scores by health insurance features. More specifically, the coefficients of linear regressions were used in post-estimation to calculate the unadjusted and adjusted means with 95% confidence intervals (95% CI) for each level or category of a dependent variable. Effect sizes (Cohen's *d*) were used to estimate the magnitude of the difference between the reference mean and the other mean levels (Cohen, 1988). Cohen's *d* can have either a negative or a positive value. A Cohen's *d* of 0.2 is considered a small effect, 0.5 is a medium effect, and a large effect is ≥0.8 and higher.

Generalized linear models were used to examine the associations between HIL, health insurance features, and the likelihood of having medical debt. The effects of interactions between HIL and health insurance features were also examined. Relative risk or risk ratio (RR) with 95% confidence intervals (95% CI) were presented for better interpretation of the results. In addition, given that HIL was modeled as a continuous variable, we calculated predictive margins to see how the probability of having medical debt differs with HIL score (Williams, 2012). Predicted probabilities were calculated at HIL scores of 7, 14, 21, and 28. A *p* value of < .05 was considered significant for all statistical tests. Multicollinearity among the independent variables was assessed using the “collin” command. The variance inflation factor (VIF) values were less than 2. A VIF of 10 is viewed as problematic. Tolerance values were not lower than 0.1, which is similar to having a VIF of 10. All statistical analyses were conducted using STATA software (Version 16.0) and accounted for the complex sampling design of the HRMS (Holahan & Long, 2017, 2019; Long et al., 2014).

## Results

**Table 1** shows sample characteristics stratified by medical debt (i.e., medical bills that were being paid over time). Overall, 16.17% of the sample had medical bills that were being paid over time. The majority of the sample (70.34%) had private insurance coverage, and 42.68% reported out-of-pocket health care costs of $1,000 or higher that were not covered by their health insurance. With higher scores indicating higher HIL, the total HIL average score (out of a possible 28) was 23.30 ± 4.87. HIL scores were lower among people with medical debt compared to those without medical debt (21.69 ± 5.59 vs. 23.79 ± 4.65, *p* < .001). Medical debt was more prevalent among people with public insurance coverage, an annual deductible of $2,500 and higher, and out-of-pocket health care costs of $1,000 or higher. Medical debt was also higher among people with less than a high school education, income ≤399% of the federal poverty level, and in poor/fair health.

**Table 1:**
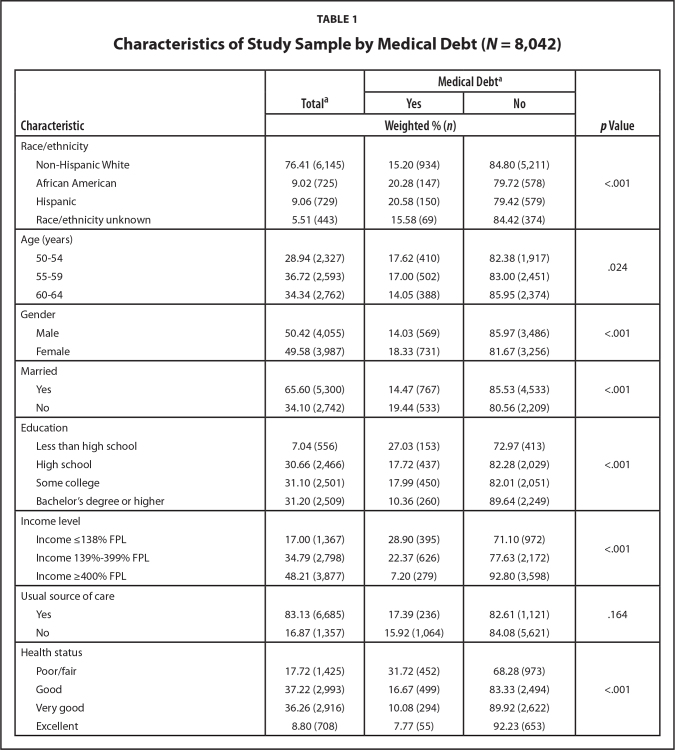

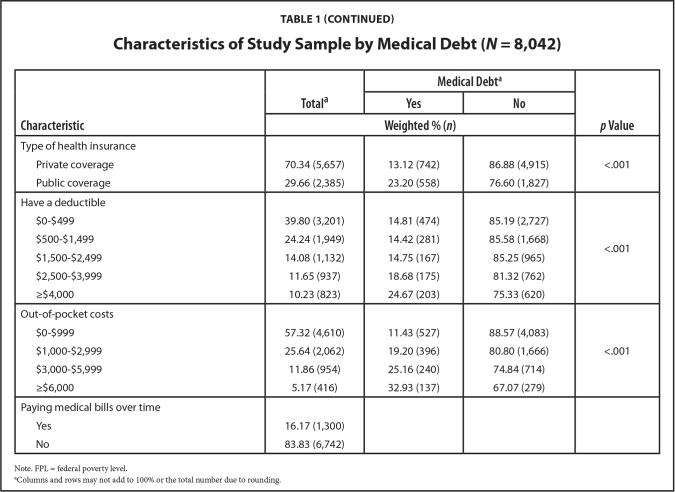
Characteristics of Study Sample by Medical Debt (*N* = 8,042)

**Characteristic**	**Total^a^**	**Medical Debt^a^**		***p* Value**

		**Yes**	**No**	

	**Weighted % (*n*)**			

Race/ethnicity				
Non-Hispanic White	76.41 (6,145)	15.20 (934)	84.80 (5,211)	<.001
African American	9.02 (725)	20.28 (147)	79.72 (578)	
Hispanic	9.06 (729)	20.58 (150)	79.42 (579)	
Race/ethnicity unknown	5.51 (443)	15.58 (69)	84.42 (374)	

Age (years)				
50–54	28.94 (2,327)	17.62 (410)	82.38 (1,917)	.024
55–59	36.72 (2,593)	17.00 (502)	83.00 (2,451)	
60–64	34.34 (2,762)	14.05 (388)	85.95 (2,374)	

Gender				
Male	50.42 (4,055)	14.03 (569)	85.97 (3,486)	<.001
Female	49.58 (3,987)	18.33 (731)	81.67 (3,256)	

Married				
Yes	65.60 (5,300)	14.47 (767)	85.53 (4,533)	<.001
No	34.10 (2,742)	19.44 (533)	80.56 (2,209)	

Education				
Less than high school	7.04 (556)	27.03 (153)	72.97 (413)	<.001
High school	30.66 (2,466)	17.72 (437)	82.28 (2,029)	
Some college	31.10 (2,501)	17.99 (450)	82.01 (2,051)	
Bachelor's degree or higher	31.20 (2,509)	10.36 (260)	89.64 (2,249)	

Income level				
Income ≤138% FPL	17.00 (1,367)	28.90 (395)	71.10 (972)	<.001
Income 139%–399% FPL	34.79 (2,798)	22.37 (626)	77.63 (2,172)	
Income ≥400% FPL	48.21 (3,877)	7.20 (279)	92.80 (3,598)	

Usual source of care				
Yes	83.13 (6,685)	17.39 (236)	82.61 (1,121)	.164
No	16.87 (1,357)	15.92 (1,064)	84.08 (5,621)	

Health status				
Poor/fair	17.72 (1,425)	31.72 (452)	68.28 (973)	<.001
Good	37.22 (2,993)	16.67 (499)	83.33 (2,494)	
Very good	36.26 (2,916)	10.08 (294)	89.92 (2,622)	
Excellent	8.80 (708)	7.77 (55)	92.23 (653)	

Type of health insurance				
Private coverage	70.34 (5,657)	13.12 (742)	86.88 (4,915)	<.001
Public coverage	29.66 (2,385)	23.20 (558)	76.60 (1,827)	

Have a deductible				
$0–$499	39.80 (3,201)	14.81 (474)	85.19 (2,727)	<.001
$500–$1,499	24.24 (1,949)	14.42 (281)	85.58 (1,668)	
$1,500–$2,499	14.08 (1,132)	14.75 (167)	85.25 (965)	
$2,500–$3,999	11.65 (937)	18.68 (175)	81.32 (762)	
≥$4,000	10.23 (823)	24.67 (203)	75.33 (620)	

Out-of-pocket costs				
$0–$999	57.32 (4,610)	11.43 (527)	88.57 (4,083)	<.001
$1,000–$2,999	25.64 (2,062)	19.20 (396)	80.80 (1,666)	
$3,000–$5,999	11.86 (954)	25.16 (240)	74.84 (714)	
≥$6,000	5.17 (416)	32.93 (137)	67.07 (279)	

Paying medical bills over time				
Yes	16.17 (1,300)			
No	83.83 (6,742)			

Note. FPL = federal poverty level.

a Columns and rows may not add to 100% or the total number due to rounding.

**Table 2** shows the unadjusted and adjusted mean scores (with 95% CI) of HIL by all study variables. The unadjusted mean HIL scores were significantly lower with public versus private insurance (22.27 vs. 23.94, *p* ≤ .005, *d* = −0.35). When adjusted for all covariates, the significant difference observed in HIL by type of insurance coverage became insignificant (*d* = −0.02). The unadjusted and adjusted mean differences observed (i.e., effect sizes) for out-of-pocket costs and annual deductible were small (<0.2). Adjusted HIL scores were significantly lower among those with lower education, lower income, and poorer health when compared to their reference. The effect size estimations (Cohen's *d*) ranged from 0.29 to 0.55 (i.e., small to medium).

**Table 2:**
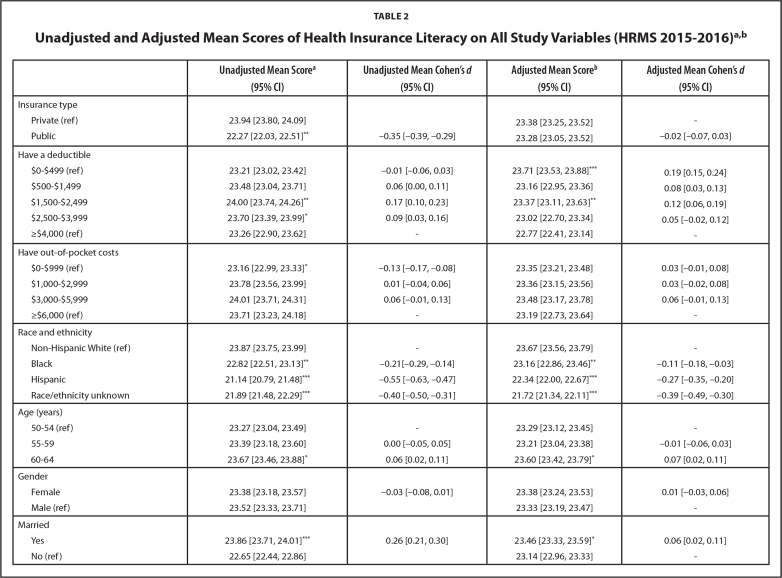

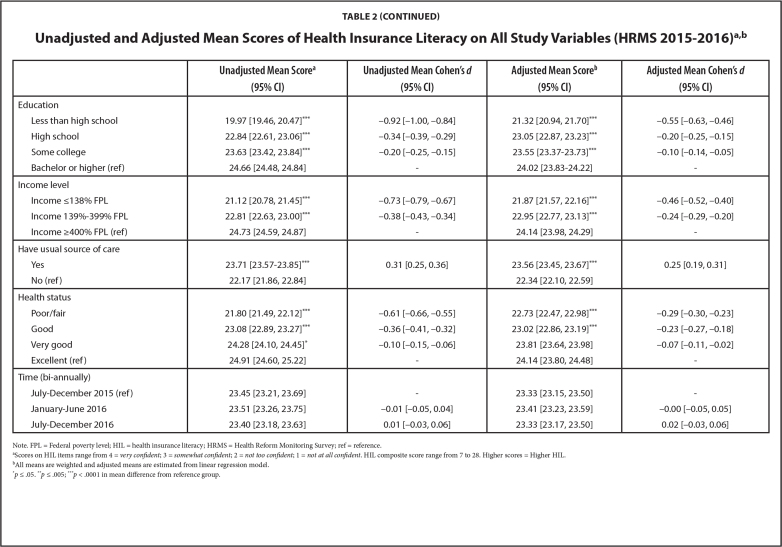
Unadjusted and Adjusted Mean Scores of Health Insurance Literacy on All Study Variables (HRMS 2015–2016)^a,b^

	**Unadjusted Mean Score^a^ (95% CI)**	**Unadjusted Mean Cohen's *d *(95% CI)**	**Adjusted Mean Score^b^ (95% CI)**	**Adjusted Mean Cohen's *d *(95% CI)**

Insurance type				
Private (ref)	23.94 [23.80, 24.09]		23.38 [23.25, 23.52]	-
Public	22.27 [22.03, 22.51]^**^	−0.35 [−0.39, −0.29]	23.28 [23.05, 23.52]	−0.02 [−0.07, 0.03]

Have a deductible				
$0–$499 (ref)	23.21 [23.02, 23.42]	−0.01 [−0.06, 0.03]	23.71 [23.53, 23.88]^***^	0.19 [0.15, 0.24]
$500–$1,499	23.48 [23.04, 23.71]	0.06 [0.00, 0.11]	23.16 [22.95, 23.36]	0.08 [0.03, 0.13]
$1,500–$2,499	24.00 [23.74, 24.26]^**^	0.17 [0.10, 0.23]	23.37 [23.11, 23.63]^**^	0.12 [0.06, 0.19]
$2,500–$3,999	23.70 [23.39, 23.99]^*^	0.09 [0.03, 0.16]	23.02 [22.70, 23.34]	0.05 [−0.02, 0.12]
≥$4,000 (ref)	23.26 [22.90, 23.62]	-	22.77 [22.41, 23.14]	-

Have out-of-pocket costs				
$0–$999 (ref)	23.16 [22.99, 23.33]^*^	−0.13 [−0.17, −0.08]	23.35 [23.21, 23.48]	0.03 [−0.01, 0.08]
$1,000–$2,999	23.78 [23.56, 23.99]	0.01 [−0.04, 0.06]	23.36 [23.15, 23.56]	0.03 [−0.02, 0.08]
$3,000–$5,999	24.01 [23.71, 24.31]	0.06 [−0.01, 0.13]	23.48 [23.17, 23.78]	0.06 [−0.01, 0.13]
≥$6,000 (ref)	23.71 [23.23, 24.18]	-	23.19 [22.73, 23.64]	-

Race and ethnicity				
Non-Hispanic White (ref)	23.87 [23.75, 23.99]	-	23.67 [23.56, 23.79]	-
Black	22.82 [22.51, 23.13]^**^	−0.21[−0.29, −0.14]	23.16 [22.86, 23.46]^**^	−0.11 [−0.18, −0.03]
Hispanic	21.14 [20.79, 21.48]^***^	−0.55 [−0.63, −0.47]	22.34 [22.00, 22.67]^***^	−0.27 [−0.35, −0.20]
Race/ethnicity unknown	21.89 [21.48, 22.29]^***^	−0.40 [−0.50, −0.31]	21.72 [21.34, 22.11]^***^	−0.39 [−0.49, −0.30]

Age (years)				
50–54 (ref)	23.27 [23.04, 23.49]	-	23.29 [23.12, 23.45]	-
55–59	23.39 [23.18, 23.60]	0.00 [−0.05, 0.05]	23.21 [23.04, 23.38]	−0.01 [−0.06, 0.03]
60–64	23.67 [23.46, 23.88]^*^	0.06 [0.02, 0.11]	23.60 [23.42, 23.79]^*^	0.07 [0.02, 0.11]

Gender				
Female	23.38 [23.18, 23.57]	−0.03 [−0.08, 0.01]	23.38 [23.24, 23.53]	0.01 [−0.03, 0.06]
Male (ref)	23.52 [23.33, 23.71]		23.33 [23.19, 23.47]	-

Married				
Yes	23.86 [23.71, 24.01]^***^	0.26 [0.21, 0.30]	23.46 [23.33, 23.59]^*^	0.06 [0.02, 0.11]
No (ref)	22.65 [22.44, 22.86]		23.14 [22.96, 23.33]	-

Education				
Less than high school	19.97 [19.46, 20.47]^***^	−0.92 [−1.00, −0.84]	21.32 [20.94, 21.70]^***^	−0.55 [−0.63, −0.46]
High school	22.84 [22.61, 23.06]^***^	−0.34 [−0.39, −0.29]	23.05 [22.87, 23.23]^***^	−0.20 [−0.25, −0.15]
Some college	23.63 [23.42, 23.84]^***^	−0.20 [−0.25, −0.15]	23.55 [23.37–23.73]^***^	−0.10 [−0.14, −0.05]
Bachelor or higher (ref)	24.66 [24.48, 24.84]	-	24.02 [23.83–24.22]	-

Income level				
Income ≤138% FPL	21.12 [20.78, 21.45]^***^	−0.73 [−0.79, −0.67]	21.87 [21.57, 22.16]^***^	−0.46 [−0.52, −0.40]
Income 139%–399% FPL	22.81 [22.63, 23.00]^***^	−0.38 [−0.43, −0.34]	22.95 [22.77, 23.13]^***^	−0.24 [−0.29, −0.20]
Income ≥400% FPL (ref)	24.73 [24.59, 24.87]	-	24.14 [23.98, 24.29]	-

Have usual source of care				
Yes	23.71 [23.57–23.85]^***^	0.31 [0.25, 0.36]	23.56 [23.45, 23.67]^***^	0.25 [0.19, 0.31]
No (ref)	22.17 [21.86, 22.84]		22.34 [22.10, 22.59]	

Health status				
Poor/fair	21.80 [21.49, 22.12]^***^	−0.61 [−0.66, −0.55]	22.73 [22.47, 22.98]^***^	−0.29 [−0.30, −0.23]
Good	23.08 [22.89, 23.27]^***^	−0.36 [−0.41, −0.32]	23.02 [22.86, 23.19]^***^	−0.23 [−0.27, −0.18]
Very good	24.28 [24.10, 24.45]^*^	−0.10 [−0.15, −0.06]	23.81 [23.64, 23.98]	−0.07 [−0.11, −0.02]
Excellent (ref)	24.91 [24.60, 25.22]	-	24.14 [23.80, 24.48]	-

Time (bi-annually)				
July–December 2015 (ref)	23.45 [23.21, 23.69]	-	23.33 [23.15, 23.50]	-
January–June 2016	23.51 [23.26, 23.75]	−0.01 [−0.05, 0.04]	23.41 [23.23, 23.59]	−0.00 [−0.05, 0.05]
July–December 2016	23.40 [23.18, 23.63]	0.01 [−0.03, 0.06]	23.33 [23.17, 23.50]	0.02 [−0.03, 0.06]

Note. FPL = Federal poverty level; HIL = health insurance literacy; HRMS = Health Reform Monitoring Survey; ref = reference.

a Scores on HIL items range from 4 = *very confident*; 3 = *somewhat confident*; 2 = *not too confident*; 1 = *not at all confident*. HIL composite score range from 7 to 28. Higher scores = Higher HIL.

b All means are weighted and adjusted means are estimated from linear regression model.

* *p*≤ .05.

** *p*≤ .005;

*** *p*< .0001 in mean difference from reference group.

**Figure 2.**
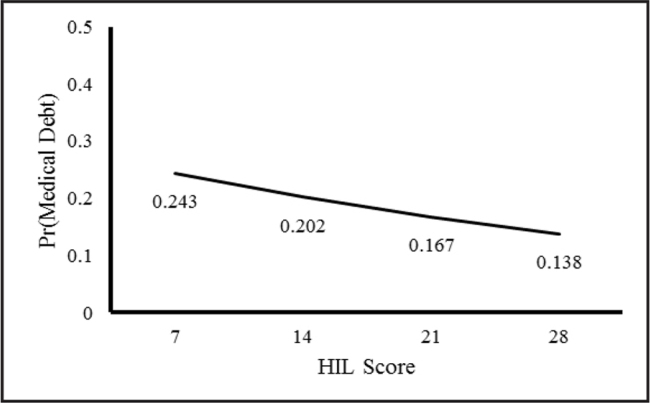
Mean HIL score by probability of having medical debt: Health Reform Monitoring Survey. Figure generated from the generalized linear model results. HIL = health insurance literacy. Pr = probability.

**Table 3** shows the results from the generalized linear models assessing the associations between HIL, health insurance features, and medical debt. Unadjusted (Model 1), higher HIL was associated with a 6% lower relative risk of having medical bills that are being paid over time (RR = 0.94; 95% CI [0.93, 0.95]). Adjusting for health insurance, annual deductible, and out-of-pocket costs (Models 2 to 4) lowered the risk of having medical bills to 5% (RR = 0.95; 95% CI [0.94, 0.95]). With the inclusion of income and education (Models 5 & 6), the risk of having medical bills decreased by an additional 2% (RR = 0.97; 95% CI [0.96, 0.98]). Based on the predictive margins calculated from Model 6 (**Figure 2**), a person with a mean score of 7 had a 24.3% risk of having medical bills that are being paid over time, while a person with a score of 28 had a 13.8% risk.

**Table 3:**
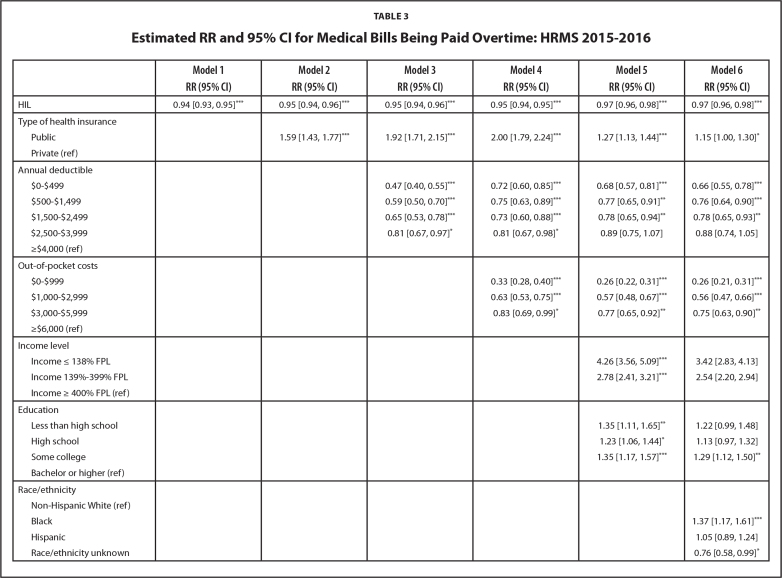

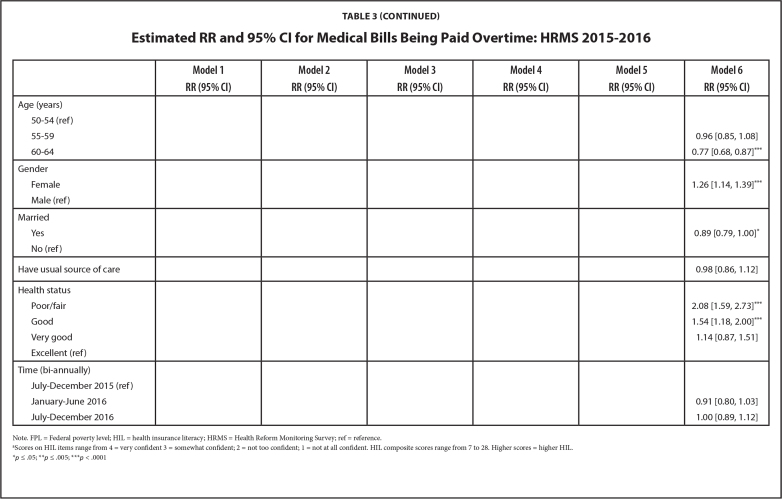
Estimated RR and 95% CI for Medical Bills Being Paid Overtime: HRMS 2015–2016

	**Model 1 RR (95% CI)**	**Model 2 RR (95% CI)**	**Model 3 RR (95% CI)**	**Model 4 RR (95% CI)**	**Model 5 RR (95% CI)**	**Model 6 RR (95% CI)**

HIL	0.94 [0.93, 0.95]^***^	0.95 [0.94, 0.96]^***^	0.95 [0.94, 0.96]^***^	0.95 [0.94, 0.95]^***^	0.97 [0.96, 0.98]^***^	0.97 [0.96, 0.98]^***^

Type of health insurance						
Public		1.59 [1.43, 1.77]^***^	1.92 [1.71, 2.15]^***^	2.00 [1.79, 2.24]^***^	1.27 [1.13, 1.44]^***^	1.15 [1.00, 1.30]^*^
Private (ref)						

Annual deductible						
$0–$499			0.47 [0.40, 0.55]^***^	0.72 [0.60, 0.85]^***^	0.68 [0.57, 0.81]^***^	0.66 [0.55, 0.78]^***^
$500–$1,499			0.59 [0.50, 0.70]^***^	0.75 [0.63, 0.89]^***^	0.77 [0.65, 0.91]^**^	0.76 [0.64, 0.90]^***^
$1,500–$2,499			0.65 [0.53, 0.78]^***^	0.73 [0.60, 0.88]^***^	0.78 [0.65, 0.94]^**^	0.78 [0.65, 0.93]^**^
$2,500–$3,999			0.81 [0.67, 0.97]^*^	0.81 [0.67, 0.98]^*^	0.89 [0.75, 1.07]	0.88 [0.74, 1.05]
≥$4,000 (ref)						

Out-of-pocket costs						
$0–$999				0.33 [0.28, 0.40]^***^	0.26 [0.22, 0.31]^***^	0.26 [0.21, 0.31]^***^
$1,000–$2,999				0.63 [0.53, 0.75]^***^	0.57 [0.48, 0.67]^***^	0.56 [0.47, 0.66]^***^
$3,000–$5,999				0.83 [0.69, 0.99]^*^	0.77 [0.65, 0.92]^**^	0.75 [0.63, 0.90]^**^
≥$6,000 (ref)						

Income level						
Income ≤ 138% FPL					4.26 [3.56, 5.09]^***^	3.42 [2.83, 4.13]
Income 139%–399% FPL					2.78 [2.41, 3.21]^***^	2.54 [2.20, 2.94]
Income ≥ 400% FPL (ref)						

Education						
Less than high school					1.35 [1.11, 1.65]^**^	1.22 [0.99, 1.48]
High school					1.23 [1.06, 1.44]^*^	1.13 [0.97, 1.32]
Some college					1.35 [1.17, 1.57]^***^	1.29 [1.12, 1.50]^**^
Bachelor or higher (ref)						

Race/ethnicity						
Non-Hispanic White (ref)						
Black						1.37 [1.17, 1.61]^***^
Hispanic						1.05 [0.89, 1.24]
Race/ethnicity unknown						0.76 [0.58, 0.99]^*^

Age (years)						
50–54 (ref)						
55–59						0.96 [0.85, 1.08]
60–64						0.77 [0.68, 0.87]^***^

Gender						
Female						1.26 [1.14, 1.39]^***^
Male (ref)						

Married						
Yes						0.89 [0.79, 1.00]^*^
No (ref)						

Have usual source of care						0.98 [0.86, 1.12]

Health status						
Poor/fair						2.08 [1.59, 2.73]^***^
Good						1.54 [1.18, 2.00]^***^
Very good						1.14 [0.87, 1.51]
Excellent (ref)						

Time (bi-annually)						
July–December 2015 (ref)						
January–June 2016						0.91 [0.80, 1.03]
July–December 2016						1.00 [0.89, 1.12]

Note. FPL = Federal poverty level; HIL = health insurance literacy; HRMS = Health Reform Monitoring Survey; ref = reference.

a Scores on HIL items range from 4 = very confident 3 = somewhat confident; 2 = not too confident; 1 = not at all confident. HIL composite scores range from 7 to 28. Higher scores = higher HIL.

* *p*≤ .05;

** *p*≤ .005;

*** *p*< .0001

Adjusted for HIL (Model 2), people with public insurance coverage had a higher risk of having medical bills that are being paid over time than those with private coverage (RR = 1.59; 95% CI [1.43, 1.77]). Adjusting for annual deductible and out-of-pocket costs (Model 4) increased the risk of having medical bills that are being paid over time for the publicly insured (RR = 2.00; 95% CI [1.79, 2.24]). However, accounting for income, education, and health status (Model 5 & 6) lowered the risk of having medical bills that are being paid over time for the publicly insured (RR = 1.27; 95% CI [1.13, 1.44] and RR = 1.15; 95% CI [1.00, 1.30], respectively). The risk of having medical bills that are being paid over time increases with higher annual deductibles and out-of-pocket costs (Models 5 & 6). There were no significant interactions effects between HIL and health insurance features on medical debt. The risks of having medical debt were higher with lower income and poorer health.

## Discussion

We examined the relationships between HIL, health insurance features, and medical debt in a national, internet-based sample of middle-age Americans. We found that adjusted mean HIL scores did not differ significantly by insurance type (private vs. public) or out-of-pocket costs. Mean HIL scores were lower with higher deductibles; however, differences in mean scores were small. Higher HIL was associated with lower risk of having medical debt. Publicly insured people had a higher risk of medical debt than the privately insured. Higher annual deductibles and out-of-pocket costs were associated with higher risk of having medical debt.

Unlike previous research (Kutner et al., 2006; Loewenstein et al., 2013), HIL did not appear to differ by type of insurance coverage or by out-of-pocket-costs. People with public insurance coverage have been shown to have lower HIL than those with private insurance (Kutner et al., 2006; Loewenstein et al., 2013). Our contrary findings may be due to the age and education of people in our study population. Over 60% of our internet-based sample had some college education or higher, suggesting that this internet-based sample may be savvier and familiar with the features (e.g., copays and deductible) of insurance coverage. Moreover, 70% of people age 50 to 64 years have at least one chronic health condition, which requires regular contact with the health care system (Centers for Disease Control and Prevention, 2009), and therefore likely know how health insurance works. The percentage of non-Hispanic White people age 50 to 64 years in our HRMS sample was also higher than the national average. White people are more likely to be insured and have regular contact with the health care system than racial/ethnic minorities (Sohn, 2017).

The small difference in HIL by annual deductible may be a reflection of the knowledge and experiences of newly insured people who tend to have problems navigating the health care system and health insurance networks (Garfield & Young, 2015). Newly insured people are often younger, less educated, have poor HIL, and tend to enroll in high cost-sharing plans (Bhargava et al., 2017; Garfield & Young, 2015; O'Connor & Kabadayi, 2020). They also use more and inappropriate health care services (Finkelstein et al., 2016).

Our finding that higher HIL correlated with lower medical debt among middle-age Americans is consistent with the literature suggesting that HIL plays an important role in the financial hardship of vulnerable populations (McCormack et al., 2009; Zhao et al., 2019). For example, Zhao et al. (2019) found that financial hardship among cancer patients was associated with poor HIL, which may have limited their ability to avoid higher drug costs and navigate options in the health care system. Poor HIL is only one of the impediments to the effective use of health care services and navigation of the health care system (Braga et al., 2017). Our findings also suggest that HIL is important in reducing, not preventing, the likelihood of having medical debt. At the highest HIL score, we found that people still had a 13.8% risk of having medical debt. Braga et al. (2017) found that while people with financial knowledge had a lower risk of past-due medical debt, financial education did not reduce the likelihood of having past-due medical debt.

Type of insurance, annual deductible, and out-of-pocket health care costs predicted medical debt. It is unsurprising that the publicly insured and those with higher deductibles and out-of-pocket costs are more at risk of medical debt. While the ACA expansions of public insurance coverage (i.e., Medicaid) have significantly reduced the number of unpaid bills and the amount of debt among low-income people (Hu et al., 2018), public insurance coverage does not cover all the health care costs or services needed (Collins, Bhupal, et al., 2019). High deductibles and out-of-pocket health care costs also cause people to be underinsured and susceptible to medical bill and debt problems (Collins, Bhupal, et al., 2019). It is well documented that people in high cost-sharing health plans have higher out-of-pocket expenses and are more likely to incur debt than those in traditional plans (Collins, Bhupal, et al., 2019; Galbraith et al., 2011). With increasing cost-sharing in private plans, the challenges in affordability are becoming similar for people in private and public health insurance plans (Collins, Bhupal, et al., 2019; Collins, Radley, et al., 2019).

This study has limitations that should be noted. First, the data is self-reported, which is subject to recall bias. Second, the internet-based survey may result in some groups (e.g., people with low-income and people who are undereducated) being underrepresented due to inequitable access to the internet and computers. Third, the data were cross-sectional, making causality hard to establish. Nonetheless, this study provides important insight into HIL and medical debt among middle-age Americans, a population that tends to have high health care needs and incur high health care costs (Yee et al., 2012). It also contributes to the limited literature on the importance of HIL to health care use and outcomes (Paez et al., 2014).

Our study findings have implications for policy and research relating to HIL and medical debt. While HIL promotes effective use of health care services and insurance benefits, it does eliminate the likelihood of having medical debt. Research shows that a majority of middle-age Americans (67.7%) are concerned about changes in federal policies pertaining to health insurance (Tipirneni et al., 2020). In addition to interventions to improve Americans' HIL, effective policies to address systems factors such as the complexity of insurance plan features and choices, affordability, and coverage of health care services are needed to eliminate medical bill problems and debt (Schoen et al., 2013). Additional research is needed to better understand HIL among high health care needs and costs people and whether improving HIL alleviates their medical bill problems and debt.

## References

[x24748307-20211102-01-bibr1] Andersen , R. M. ( 1995 ). Revisiting the behavioral model and access to medical care: Does it matter? *Journal of Health and Social Behavior* , *36* ( 1 ), 1 – 10 . 10.2307/2137284 PMID: 7738325

[x24748307-20211102-01-bibr2] Andersen , R. , & Newman , J. F. ( 2005 ). Societal and individual determinants of medical care utilization in the United States . *The Milbank Quarterly* , *83* ( 4 ). 10.1111/j.1468-0009.2005.00428.x 4198894

[x24748307-20211102-01-bibr3] Bartholomae , S. , Russell , M. B. , Braun , B. , & McCoy , T. ( 2016 ). Building health insurance literacy: Evidence from the Smart Choice Health Insurance (TM) program . *Journal of Family and Economic Issues* , *37* ( 2 ), 140 – 155 . 10.1007/s10834-016-9482-7

[x24748307-20211102-01-bibr4] Bhargava , S. , Loewenstein , G. , & Sydnor , J. ( 2017 ). Choose to lose: Health plan choices from a menu with dominated options . *The Quarterly Journal of Economics* , *132* ( 3 ), 1319 – 1372 . 10.1093/qje/qjx011

[x24748307-20211102-01-bibr5] Braga , B. , McKernan , S. M. , & Karas , A . ( 2017 , March ). *Is financial knowledge associated with past-due medical debt?* Urban Institute . https://www.urban.org/sites/default/files/publication/88591/financial_knowledge_associated_with_past_due_medical_debt.pdf

[x24748307-20211102-01-bibr6] Centers for Disease Control and Prevention . ( 2009 ). *Promoting preventive services for adults 50–64: Community and clinical partnerships* . https://www.cdc.gov/aging/agingdata/data-portal/preventive-services.html

[x24748307-20211102-01-bibr7] Chen , L.-W. , Zhang , W. , Meza , J. , Fraser , R. , Mueller , K. J. , Adidam , P. T. , Pol , L. , & Shea , D. G. ( 2004 ). *Pent-up demand: Health care use of the uninsured near elderly* . Economic Research Initiative on the Uninsured . http://www.rwjf-eriu.org/pdf/wp26.pdf

[x24748307-20211102-01-bibr8] Cohen , J . ( 1988 ). *Statistical Power Analysis for the Behavioral Sciences* ( 2nd ed. ). Lawrence Erlbaum Associates .

[x24748307-20211102-01-bibr9] Collins , S. R. , Bhupal , H. K. , & Doty , M. M. ( 2019 ). *Health insurance coverage eight years after the ACA: Fewer uninsured Americans and shorter coverage gaps, but more underinsured* . Commonwealth Fund . https://www.commonwealthfund.org/publications/issue-briefs/2019/feb/health-insurance-coverage-eight-years-after-aca

[x24748307-20211102-01-bibr10] Collins , S. R. , Radley , D. C. , & Baumgartner , J. C. ( 2019 ). *Trends in employer health care coverage, 2008–2018: Higher costs for workers and their families* . Commonwealth Fund . https://www.commonwealthfund.org/publications/2019/nov/trends-employer-health-care-coverage-2008-2018

[x24748307-20211102-01-bibr11] Doty , M. M. , Collins , S. R. , Rustgi , S. D. , & Kriss , J. L. ( 2008 ). *Seeing red: The growing burden of medical bills and debt faced by U.S. families* . Commonwealth Fund . https://www.commonwealthfund.org/publications/issue-briefs/2008/aug/seeing-red-growing-burden-medical-bills-and-debt-faced-us PMID: 19798802

[x24748307-20211102-01-bibr12] Finkelstein , A. N. , Taubman , S. L. , Allen , H. L. , Wright , B. J. , & Baicker , K. ( 2016 ). Effect of Medicaid coverage on ED use—Further evidence from Oregon's experiment . *The New England Journal of Medicine* , *375* ( 16 ), 1505 – 1507 . 10.1056/NEJMp1609533 PMID: 27797307

[x24748307-20211102-01-bibr13] Galbraith , A. A. , Ross-Degnan , D. , Soumerai , S. B. , Rosenthal , M. B. , Gay , C. , & Lieu , T. A. ( 2011 ). Nearly half of families in high-deductible health plans whose members have chronic conditions face substantial financial burden . *Health Affairs (Project Hope)* , *30* ( 2 ), 322 – 331 . 10.1377/hlthaff.2010.0584 PMID: 21289354 PMC4423400

[x24748307-20211102-01-bibr14] Garfield , R. , & Young , K . ( 2015 ). *How does gaining coverage affect people's lives? Access, utilization, and financial security among newly insured adults* . https://www.kff.org/health-reform/issue-brief/how-does-gaining-coverage-affect-peoples-lives-access-utilization-and-financial-security-among-newly-insured-adults/view/print/

[x24748307-20211102-01-bibr15] Himmelstein , D. U. , Thorne , D. , Warren , E. , & Woolhandler , S. ( 2009 ). Medical bankruptcy in the United States, 2007: Results of a national study . *The American Journal of Medicine* , *122* ( 8 ), 741 – 746 . 10.1016/j.amjmed.2009.04.012 PMID: 19501347

[x24748307-20211102-01-bibr16] Holahan , J. , & Long , S. K. ( 2017 ). *Health Reform Monitoring Survey, United States, first quarter 2013* . https://www.icpsr.umich.edu/web/HMCA/studies/35624

[x24748307-20211102-01-bibr17] Holahan , J. , & Long , S. K. ( 2019 ). *Health Reform Monitoring Survey, United States, third quarter 2016* . https://www.icpsr.umich.edu/web/HMCA/studies/36842

[x24748307-20211102-01-bibr18] Hu , L. , Kaestner , R. , Mazumder , B. , Miller , S. , & Wong , A. ( 2018 ). The effect of the affordable care act Medicaid expansions on financial wellbeing . *Journal of Public Economics* , *163* , 99 – 112 . 10.1016/j.jpubeco.2018.04.009 PMID: 30393411 PMC6208351

[x24748307-20211102-01-bibr19] Jones , D. K. , Gusmano , M. K. , Nadash , P. , & Miller , E. A. ( 2018 ). Undermining the ACA through the executive branch and federalism: What the Trump administration's approach to health reform means for older Americans . *Journal of Aging & Social Policy* , *30* ( 3–4 ), 282 – 299 . 10.1080/08959420.2018.1462684 PMID: 29649407

[x24748307-20211102-01-bibr20] Komisar , H. L. ( 2012 ). *Key issues in understanding the economic and health security of current and future generations of seniors* . Henry J. Kaiser Family Foundation . https://www.kff.org/wp-content/uploads/2013/01/8289.pdf

[x24748307-20211102-01-bibr21] Komisar , H. L. ( 2013 ). *The effects of rising health care costs on middle-class economic security* . AARP Public Policy Institute . http://www.aarp.org/content/dam/aarp/research/public_policy_institute/security/2013/impact-of-rising-healthcare-costs-AARP-ppi-sec.pdf

[x24748307-20211102-01-bibr22] Kullgren , J. T. , Galbraith , A. A. , Hinrichsen , V. L. , Miroshnik , I. , Penfold , R. B. , Rosenthal , M. B. , Landon , B. E. , & Lieu , T. A. ( 2010 ). Health care use and decision making among lower-income families in high-deductible health plans . *Archives of Internal Medicine* , *170* ( 21 ), 1918 – 1925 . 10.1001/archinternmed.2010.428 PMID: 21098352 PMC4004054

[x24748307-20211102-01-bibr23] Kutner , M. , Greenburg , E. , Jin , Y. , & Paulsen , C . ( 2006 ). *The health literacy of America's adults: Results from the 2003 National Assessment of Adult Literacy* . U.S. Department of Education . https://nces.ed.gov/pubsearch/pubsinfo.asp?pubid=2006483

[x24748307-20211102-01-bibr24] Loewenstein , G. , Friedman , J. Y. , McGill , B. , Ahmad , S. , Linck , S. , Sinkula , S. , Beshears , J. , Choi , J. J. , Kolstad , J. , Laibson , D. , Madrian , B. C. , List , J. A. , & Volpp , K. G. ( 2013 ). Consumers' misunderstanding of health insurance . *Journal of Health Economics* , *32* ( 5 ), 850 – 862 . 10.1016/j.jhealeco.2013.04.004 PMID: 23872676

[x24748307-20211102-01-bibr25] Long , S. K. , Kenney , G. M. , Zuckerman , S. , Goin , D. E. , Wissoker , D. , Blavin , F. , Blumberg , L. J. , Clemans-Cope , L. , Holahan , J. , & Hempstead , K. ( 2014 ). The health reform monitoring survey: Addressing data gaps to provide timely insights into the affordable care act . *Health Affairs (Project Hope)* , *33* ( 1 ), 161 – 167 . 10.1377/hlthaff.2013.0934 PMID: 24352654

[x24748307-20211102-01-bibr26] McCormack , L. , Bann , C. , Uhrig , J. , Berkman , N. , & Rudd , R. ( 2009 ). Health insurance literacy of older adults . *The Journal of Consumer Affairs* , *43* ( 2 ), 223 – 248 . 10.1111/j.1745-6606.2009.01138.x

[x24748307-20211102-01-bibr27] Morgan , R. O. , Teal , C. R. , Hasche , J. C. , Petersen , L. A. , Byrne , M. M. , Paterniti , D. A. , & Virnig , B. A. ( 2008 ). Does poorer familiarity with Medicare translate into worse access to health care? *Journal of the American Geriatrics Society* , *56* ( 11 ), 2053 – 2060 . 10.1111/j.1532-5415.2008.01993.x PMID: 19016939

[x24748307-20211102-01-bibr28] Nadash , P. , Miller , E. A. , Jones , D. K. , Gusmano , M. K. , & Rosenbaum , S. ( 2018 ). A series of unfortunate events: Implications of Republican efforts to repeal and replace the Affordable Care Act For older adults . *Journal of Aging & Social Policy* , *30* ( 3–4 ), 259 – 281 . 10.1080/08959420.2018.1462683 PMID: 29634455

[x24748307-20211102-01-bibr29] O'Connor , G. E. , & Kabadayi , S. ( 2020 ). Examining antecedents of health insurance literacy: The role of locus of control, cognitive style, and financial knowledge . *The Journal of Consumer Affairs* , *54* ( 1 ), 227 – 260 . 10.1111/joca.12266

[x24748307-20211102-01-bibr30] Paez , K. A. , Mallery , C. J. , Noel , H. , Pugliese , C. , McSorley , V. E. , Lucado , J. L. , & Ganachari , D. ( 2014 ). Development of the Health Insurance Literacy Measure (HILM): Conceptualizing and measuring consumer ability to choose and use private health insurance . *Journal of Health Communication* , *19* ( Suppl. 2 ), 225 – 239 . 10.1080/10810730.2014.936568 PMID: 25315595 PMC4200586

[x24748307-20211102-01-bibr31] Piette , J. D. , & Heisler , M. ( 2006 ). The relationship between older adults' knowledge of their drug coverage and medication cost problems . *Journal of the American Geriatrics Society* , *54* ( 1 ), 91 – 96 . 10.1111/j.1532-5415.2005.00527.x PMID: 16420203

[x24748307-20211102-01-bibr32] Rabin , D. L. , Jetty , A. , Petterson , S. , & Froehlich , A. ( 2020 ). Under the ACA higher deductibles and medical debt cause those most vulnerable to defer needed care . *Journal of Health Care for the Poor and Underserved* , *31* ( 1 ), 424 – 440 . 10.1353/hpu.2020.0031 PMID: 32037340

[x24748307-20211102-01-bibr33] Reed , M. E. , Graetz , I. , Fung , V. , Newhouse , J. P. , & Hsu , J. ( 2012 ). In consumer-directed health plans, a majority of patients were unaware of free or low-cost preventive care . *Health Affairs (Project Hope)* , *31* ( 12 ), 2641 – 2648 . 10.1377/hlthaff.2012.0059 PMID: 23213148

[x24748307-20211102-01-bibr34] Schoen , C. , Osborn , R. , Squires , D. , & Doty , M. M. ( 2013 ). Access, affordability, and insurance complexity are often worse in the United States compared to ten other countries . *Health Affairs (Project Hope)* , *32* ( 12 ), 2205 – 2215 . 10.1377/hlthaff.2013.0879 PMID: 24226092

[x24748307-20211102-01-bibr35] Sohn , H. ( 2017 ). Racial and ethnic disparities in health insurance coverage: Dynamics of gaining and losing coverage over the life-course . *Population Research and Policy Review* , *36* ( 2 ), 181 – 201 . 10.1007/s11113-016-9416-y PMID: 28366968 PMC5370590

[x24748307-20211102-01-bibr36] Sullivan , G. M. , & Artino , A. R. Jr . ( 2013 ). Analyzing and interpreting data from Likert-type scales . *Journal of Graduate Medical Education* , *5* ( 4 ), 541 – 542 . 10.4300/JGME-5-4-18 PMID: 24454995 PMC3886444

[x24748307-20211102-01-bibr37] Tipirneni , R. , Politi , M. C. , Kullgren , J. T. , Kieffer , E. C. , Goold , S. D. , & Scherer , A. M. ( 2018 ). Association between health insurance literacy and avoidance of health care services owing to cost . *JAMA Network Open* , *1* ( 7 ), e184796 Advance online publication. 10.1001/jamanetworkopen.2018.4796 PMID: 30646372 PMC6324372

[x24748307-20211102-01-bibr38] Tipirneni , R. , Solway , E. , Malani , P. , Luster , J. , Kullgren , J. T. , Kirch , M. , Singer , D. , & Scherer , A. M. ( 2020 ). Health insurance affordability concerns and health care avoidance among US adults approaching retirement . *JAMA Network Open* , *3* ( 2 ), e1920647 10.1001/jamanetworkopen.2019.20647 PMID: 32031644 PMC9578361

[x24748307-20211102-01-bibr39] Trivedi , A. N. , Moloo , H. , & Mor , V. ( 2010 ). Increased ambulatory care copayments and hospitalizations among the elderly . *The New England Journal of Medicine* , *362* ( 4 ), 320 – 328 . 10.1056/NEJMsa0904533 PMID: 20107218

[x24748307-20211102-01-bibr40] Tu , H. T. , & Liebhaber , A. B. ( 2009 ). *Rough passage: Affordable health coverage for near-elderly Americans* . Center for Studying Health System Change . https://www.nihcr.org/analysis/rough-passage/

[x24748307-20211102-01-bibr41] Turner , S. D. ( 2016 ). *Digital denied: the impact of systemic racial discrimination on home internet adoption* . Free Press .

[x24748307-20211102-01-bibr42] Williams , R. ( 2012 ). Using the margins command to estimate and interpret adjusted predictions and marginal effects . *The Stata Journal* , *12* ( 2 ), 308 – 331 . 10.1177/1536867X1201200209

[x24748307-20211102-01-bibr43] Wiltshire , J. C. , Elder , K. , & Allison , J. J. ( 2016 ). Differences in problems paying medical bills between African Americans and whites from 2007 and 2009: The underlying role of health status . *Journal of Racial and Ethnic Health Disparities* , *3* ( 2 ), 381 – 388 . 10.1007/s40615-015-0197-5 PMID: 26721765

[x24748307-20211102-01-bibr44] Wiltshire , J. C. , Elder , K. , Kiefe , C. , & Allison , J. J. ( 2016 ). Medical debt and related financial consequences among older African American and white adults . *American Journal of Public Health* , *106* ( 6 ), 1086 – 1091 . 10.2105/AJPH.2016.303137 PMID: 27077346 PMC4880274

[x24748307-20211102-01-bibr45] Yee , T. , Cunningham , P. , Jacobson , G. , Neuman , T. , & Levinson , Z . ( 2012 ). *Cost and access challenges: A comparison of experiences between uninsured and privately insured adults aged 55 to 64 with seniors on Medicare* . Kaiser Family Foundation . https://www.kff.org/wp-content/uploads/2013/01/8320.pdf

[x24748307-20211102-01-bibr46] Zhang , X. , Trish , E. , & Sood , N. ( 2018 ). Financial burden of healthcare utilization in consumer-directed health plans . *The American Journal of Managed Care* , *24* ( 4 ), e115 – e121 PMID: 29668214

[x24748307-20211102-01-bibr47] Zhao , J. , Han , X. , Zheng , Z. , Banegas , M. P. , Ekwueme , D. U. , & Yabroff , K. R. ( 2019 ). Is health insurance literacy associated with financial hardship among cancer survivors? Findings from a national sample in the United States . *JNCI Cancer Spectrum* , *3* ( 4 ), pkz061 – pkz065 . 10.1093/jncics/pkz061 PMID: 32337486 PMC7050003

[x24748307-20211102-01-bibr48] Zheng , Z. , Jemal , A. , Banegas , M. P. , Han , X. , & Yabroff , K. R. ( 2019 ). High-deductible health plans and cancer survivorship: What is the association with access to care and hospital emergency department use? *Journal of Oncology Practice/American Society of Clinical Oncology* , *15* ( 11 ), e957 – e968 . 10.1200/JOP.18.00699 PMID: 31393809

[x24748307-20211102-01-bibr49] Zilcha , I. , & Schneier , N. ( 2012 ). Out-of-pocket health expenditures: A suggested role for social security . *Risk Management & Insurance Review* , *15* ( 2 ), 153 – 164 . 10.1111/j.1540-6296.2012.01215.x

